# The Zero Suicide Model: Applying Evidence-Based Suicide Prevention Practices to Clinical Care

**DOI:** 10.3389/fpsyt.2018.00033

**Published:** 2018-02-23

**Authors:** Beth S. Brodsky, Aliza Spruch-Feiner, Barbara Stanley

**Affiliations:** ^1^Department of Psychiatry, Columbia University College of Physicians and Surgeons, New York, NY, United States; ^2^New York State Psychiatric Institute, New York, NY, United States

**Keywords:** suicide, prevention, evidence-based, psychology, interventions

## Abstract

Suicide is reaching epidemic proportions, with over 44,000 deaths by suicide in the US, and 800,000 worldwide in 2015. This, despite research and development of evidence-based interventions that target suicidal behavior directly. Suicide prevention efforts need a comprehensive approach, and research must lead to effective implementation across public and mental health systems. A 10-year systematic review of evidence-based findings in suicide prevention summarized the areas necessary for translating research into practice. These include risk assessment, means restriction, evidence-based treatments, population screening combined with chain of care, monitoring, and follow-up. In this article, we review how suicide prevention research informs implementation in clinical settings where those most at risk present for care. Evidence-based and best practices address the fluctuating nature of suicide risk, which requires ongoing risk assessment, direct intervention and monitoring. In the US, the National Action Alliance for Suicide Prevention has put forth the Zero Suicide (ZS) Model, a framework to coordinate a multilevel approach to implementing evidence-based practices. We present the Assess, Intervene and Monitor for Suicide Prevention model (AIM-SP) as a guide for implementation of ZS evidence-based and best practices in clinical settings. Ten basic steps for clinical management model will be described and illustrated through case vignette. These steps are designed to be easily incorporated into standard clinical practice to enhance suicide risk assessment, brief interventions to increase safety and teach coping strategies and to improve ongoing contact and monitoring of high-risk individuals during transitions in care and high risk periods.

Suicide is a public health crisis reaching epidemic proportions and has claimed the lives of over 44,000 individuals in the US in 2015 (1) and 800,000 people worldwide in the past year. These figures reflect an increase in death by suicide by over 25% in the US (2), and 4% internationally in the last decade (3), despite increases in multitiered suicide prevention strategies and research. A 10-year systematic review of nearly 1,800 studies (4) highlighted the importance of increasing and coordinating the application of evidence-based suicide prevention strategies and concluded that research needs to lead to implementation across public health and clinical mental health systems.

In the US, the National Action Alliance for Suicide Prevention has put forth the Zero Suicide (ZS) Model, a framework and resources to coordinate a multilevel approach to implementing evidence-based practices for suicide prevention. Founded on the principle that death by suicide is preventable for patients in behavioral health systems, the ZS model offers an integrated, system-wide strategy for suicide prevention. Four components (Identify, Engage, Treat, and Transition) address aspects of clinical care, while the other three (Lead, Train, and Improve) concern administrative approaches.

The ZS elements of clinical care dictate that systematic protocols should involve ongoing risk screening and assessment, collaborative safety planning, access to evidence-based suicide-specific care, focus on lethal means reduction, consistent engagement efforts, and support during high risk periods. We will update the current state of knowledge regarding evidence-based and best clinical practice for suicide prevention, and describe how the ZS model informs application of these practices to clinical training and practice. We present a case vignette to illustrate 10 basic steps for best practice clinical suicide management, based on the ZS model.

## The ZS Model and Clinical Training

The Assess, Intervene and Monitor for Suicide Prevention model (AIM-SP) (5) model is proposed as a framework for implementing ZS in clinical care. “Assess” refers to the use of systematic screening and comprehensive risk assessment to identify at-risk patients. “Intervene” consists of conducting suicide-specific brief and psychosocial interventions. “Monitor” provides strategies for ongoing monitoring and increased contact during known high risk periods. AIM-SP provides guidelines for clinical training and best practice in suicide prevention that can be applied in a wide range of care settings.

### Screening and Risk Assessment

Several approaches to suicide risk assessment have been developed and disseminated. The Columbia Suicide Severity Rating Scale (C-SSRS) is a validated and reliable instrument that measures current and past suicidal ideation, suicide attempts, preparatory behaviors as well as non-suicidal self-injury (NSSI), a deliberate self-harm behavior performed with no intent to die (6–8). The severity and intensity of suicidal ideation, lifetime suicide attempt and NSSI, as measured by the C-SSRS, were found to predict future suicide attempts among adolescent and young adult psychiatric emergency department (ED) patients (9). These findings contribute to the existing literature on the validity of the C-SSRS as a screening method for longitudinally predicting future suicidal behaviors (10, 11).

Other approaches consider risk factors besides suicidal ideation and behavior such as demographics, psychiatric and family history, diagnosis, trauma, and protective factors. The Suicide Assessment Five-step Evaluation and Triage (SAFE-T) (12) instrument guides clinicians to identify risk and protective factors, inquire into suicidal thoughts, plans, behavior and intent, determine risk level, and choose an appropriate intervention. SAFE-T incorporates the American Psychiatric Association Practice Guidelines for suicide assessment (13). Teaching the SAFE-T to ED nurses has been shown to enhance suicide inquiry, and increase knowledge regarding identifying risk and protective factors and determining risk level and appropriate intervention (14).

### Psychosocial Treatment Interventions

Cognitive behavior therapy (CBT) and dialectical behavior therapy (DBT) are suicide-specific psychosocial treatments with evidence base in reducing suicidality in certain populations (4, 15). Randomized controlled trials (RCTs) indicate that the most effective psychosocial treatment interventions are cognitive behavioral therapies and others with interpersonal orientations that target precipitants to self-harm (16). Brief CBT, web-based CBT, CBT-/DBT-informed family treatment and DBT are effective in reducing suicidal ideation (17); preventing the onset of suicidal ideation (18); preventing post treatment suicide attempts and reattempts (19–22); decreasing hospitalizations and ED visits; and lowering medical risk of self-injurious acts (20). DBT skills training is efficacious in reducing NSSI acts (23). In addition, the suicide-specific intervention, Collaborative Assessment and Management of Suicidality (CAMS) in comparison with treatment as usual, was found to decrease suicidal ideation and related cognitions in inpatients receiving individual therapy from CAMS-trained clinicians (24, 25). The efficacy of these specific treatment interventions may vary when applied to special high risk populations (e.g., people with schizophrenia, or prison populations).

Additional research is needed to gain knowledge regarding the specific populations in which each psychosocial treatment is most efficacious, and the components of the treatments that most effectively reduce suicide-related symptoms. Obstacles to implementation such as lack of clinician training in these approaches need to be overcome through increased implementation research and dissemination efforts.

### Brief Interventions

The safety plan intervention (SPI) (26) is a best practice brief intervention (27, 28) that incorporates evidence-based suicide risk reduction strategies such as lethal means reduction, brief problem solving and coping skills, increasing social support and identifying emergency contacts to use during a suicide crisis. In conducting a SPI, clinician and client collaborate to develop a six-step plan for staying safe. These include: identifying warning signs, individual coping skills, people and places for distraction, people to contact for help, professionals to contact for help, and steps for means safety.

Crisis response planning (29, 30) is a brief intervention (27) in which individuals use a small card to write out steps for self-identifying personal warning signs, coping strategies, enlisting social support, and accessing professional services. Within a sample of high-risk active duty soldiers, crisis response planning was found more effective than contracts for safety in preventing attempts, reducing suicide ideation and hospitalization (31).

### Lethal Means Restriction

Suicides decreased following legislation pertaining to the restriction of firearms, pesticides, barbiturate prescriptions, detoxification of domestic gas, modification of analgesics packaging, mandated use of catalytic converters in automobiles, erection of barriers at common jumping locations, lowered toxicity of antidepressants (32), and restricted access to charcoal (33). The “Access to Lethal Means” (CALM) training on strategies for talking to patients about means reduction increases gatekeeper confidence in ability to care for suicidal patients, and fosters positive changes in clinician practice. The SPI enhances clinical practice in means reduction. After receiving instructions to give the SPI to patients with positive suicide screens, nurses with no formal training were nevertheless more likely to ask about access to lethal means (34).

### Follow-up and Monitoring

The practice of contacting people and providing support after discharge from the ED or after being identified as at risk for suicide reduces suicidal behaviors and deaths (4). The Brief Intervention and Contact (BIC), a 1-hour information session and follow-up contact after ED discharge was associated with a reduced number of suicide deaths in the 18 months following discharge in a five-country RCT (35). Multidisciplinary chain-of-care networks for suicide attempters following hospitalization in Norway have resulted in lower rates of repeat attempts (36). Active contact and follow-up was found effective in preventing repeat attempts over a year following admission to the ED for suicide attempts (37), and in-person and telephone follow-ups reduced suicidal thoughts and increased hope in suicide attempters (38). In a review of 11 empirical studies of follow-up interventions (i.e., phone, postal letter, postcards, in-person, e-mail, and texting), five demonstrated significant decreases in suicidal behavior (39). A combined safety planning/structured follow-up intervention (SPI-SFU) in the VA was viewed as acceptable and helpful in preventing future suicidal behavior and promoting treatment engagement (40, 41). Social support strategies can also be employed to follow-up with and monitor individuals following suicidal behaviors. In India, a peer support intervention led to a 36% decrease in suicide attempts (42). The Attempted Suicide Short Intervention Program (ASSIP) involves numerous elements including safety planning and semistandardized letters over a span of 2 years. Results from a randomized control trial (43) suggest that ASSIP effectively reduced the risk of suicide reattempts by 80%, and led to significantly less time spent in hospitals at follow-up.

## Applying the ZS/AIM Model to Clinical Training

The evidence base provides important information regarding the interventions that can help prevent suicide. A next crucial step is to apply evidence based suicide prevention interventions to the clinical training of health and mental health professionals.

### Fluctuations in Suicide Risk

Evidence-based best practices address managing the fluctuation of suicide risk over time. A study using ecological momentary assessment (44) found that suicidal ideation, hopelessness, burdensomeness and loneliness varied considerably over the course of hours and days. Suicidal ideation has been found to recur with the emergence of depressive episodes (45). In a large community survey, suicidal ideation was reported to fluctuate irregularly prior to suicide attempt (46), and variability in suicidal ideation predicts future attempts (47).

### Gaps in Training

Despite updated guidelines for suicide prevention training in the fields of psychology, social work and psychiatry (48, 49) in the US, formal training in suicide risk assessment and management remains limited (50). There is a gap in clinical “training as usual” that needs to be filled by evidence-based clinical approaches to identify, monitor and treat fluctuations in suicide risk. For example, historically, clinical approaches have relied on the use of “safety contracts” in which clinicians ask that patients sign contracts stating that they will either not act on or reach out for help when experiencing suicidal urges. However, there is little evidence that these contracts are effective (51).

### Filling the Gap in Clinical Training

**Figure 1:**
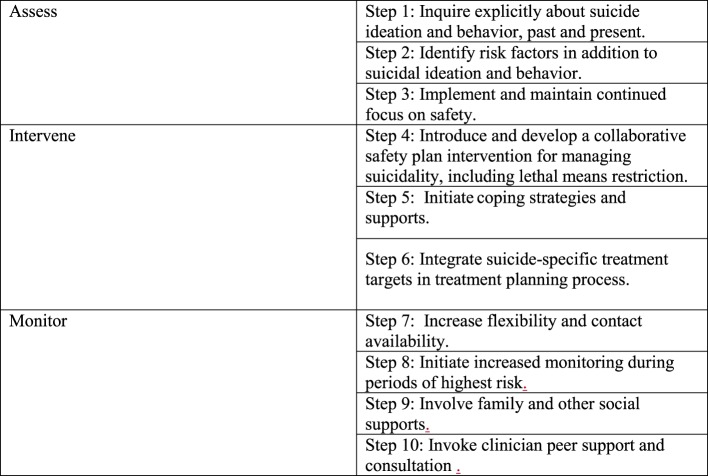
The AIM-SP model’s 10 steps for applying best suicide prevention practices to everyday clinical care.

The AIM-SP model offers 10 steps for applying best suicide prevention practices to everyday clinical care (Figure 1). We present the case of Paul to illustrate 10 basic clinical interventions for the management of suicidal behavior in an ongoing outpatient treatment, which represents only one example as to how the model informs clinical care. The Assess, Intervene and Monitor framework for suicide prevention can be applied in other settings such as inpatient or prison environments, but many require modifications of these 10 steps.

### Case Vignette

Paul is a 32-year-old single, college educated white male. He lives with a roommate and works as a graphic artist. He is talented, gets jobs easily, but has trouble keeping them. He experiences intense shame about the quality of his work. Paul periodically engages in non-suicidal self-injurious behaviors by cutting himself without suicidal intent on his upper arms with a knife. He has never tried to end his life but has had intermittent active suicidal ideation with a plan to jump from the roof of his building. On two occasions, he has gone to the roof and contemplated jumping but did not. Paul abuses alcohol, and binges on cocaine. He has aggressive episodes (e.g., gets into verbal confrontations with strangers). Paul reports being physically abused by his older brother until he was 10 years old. Paul had 3 years of outpatient therapy for depression and has been to the ED twice for NSSI behavior and active suicidal ideation. His ideation and urges to self-harm fluctuate.

### How Can the 10 Clinical Steps Be Applied to an Ongoing Outpatient Treatment with Paul?

#### Assess

Step 1: Inquire explicitly about suicidal ideation and behavior, past and present

The first step in assessing Paul’s suicide risk at any given moment is to explicitly ask whether he is having any suicidal thoughts. Paul’s clinician should not assume that he is not suicidal if he does not report it. By neglecting to ask, Paul might feel that the clinician doesn’t care or doesn’t really want to know.

Clinicians are often reluctant to ask directly. In a 2014 survey of clinicians across New York State, 20% reported discomfort in asking about suicide, and 12% would not bring up the topic of suicide even if the patient’s record or actions indicated risk (52). Clinicians feel unsure of how to intervene with someone who is at current risk for suicide, and they may erroneously believe that asking might introduce the idea.

A clinician can facilitate disclosure by building rapport and by establishing a collaborative agreement to monitor suicidal ideation. When asking directly, the clinicians should be matter-of-fact, but also warm, supportive and respectful. Knowing what to do can help the clinician balance concern with a sense of calm, to take the patient’s experiences seriously without displaying anxiety. Such an approach can facilitate open communication and possibly avoid hospitalization.

Step 2: Identify risk factors in addition to suicidal ideation and behavior

Fifty percent of those who die by suicide do so after their first and only attempt (53). Thus, in addition to fluctuating suicidal ideation, urges, suicidal and NSSI behaviors, it is important to consider non-suicide based factors that contribute to risk. The following are population based risk factors:

Demographics: male, Caucasian, age 44–65 and 85+.

- Psychiatric diagnoses: major depression, bipolar disorder, schizophrenia, BPD, PTSD, substance use, and eating disorders.
- Abuse history.
- Recent activating events: Interpersonal loss, financial, or medical problems.
- History of treatment non-adherence.
- Access to lethal means.

Protective factors: support system, religious/spiritual beliefs (e.g., that suicide is a sin), family/children, fear of dying.

Paul has no history of suicide attempts, but has two “aborted attempts” in which he started to act but stopped himself before engaging in self-harm. He also engages in NSSI behavior. Paul fits into a high risk demographic (white male entering middle age), has been diagnosed with Major Depressive Disorder and Borderline Personality Disorder, and abuses substances. He also has impulsive aggressive personality traits, a history of childhood physical abuse, and access to means (knives, rooftop, pills).

Protective factors should also be assessed. Paul is smart and talented, and likeable when not in a stormy aggressive mood. Relationships with his mother and best friend are his stated reasons for living, and he has supportive family members in his life—his uncle and cousin.

##### Risk Factors Specific to Paul—Precipitants/Recent Activating Events

For Paul, nearing a deadline on an artistic project (shame about it not being good enough and fear of exposure) is a precipitating event that can trigger suicidal ideation. Depressed mood in and of itself is NOT a risk factor for Paul’s suicidal ideation, but it does make him more vulnerable to being triggered. Increased use of alcohol and cocaine are warning signs for suicidal spikes.

Step 3: Implement and maintain continued focus on safety

Since suicidal urges fluctuate, an evidence-based clinical approach to suicide prevention necessitates ongoing assessment and continued focus on safety. Clinicians should explicitly inquire about suicidal thoughts, urges, or behaviors at each contact, and revisit and update plans for staying safe. Paul and his therapist agreed to check in about his suicidal thoughts and self-harm urges at each visit.

##### Intervene

Step 4: Introduce and develop a collaborative SPI for managing suicidality, including lethal means reduction

The SPI allows clinician and patient to develop a plan (26) for recognizing warning signs of spikes in and periods of higher risk and how to maintain safety. Safety planning increases mastery and self-efficacy for coping with suicidal urges. It can be used in both ongoing outpatient psychotherapy treatments as well as a single clinical contact such as during an ED visit. The SPI is a collaborative brief intervention that can be completed in one 30–45-min session, and then revisited/revised periodically. The six steps of the SPI are to identify: (1) warning signs, (2) internal coping strategies, (3) people and social settings that provide distraction, (4) people to contact for help, (5) professionals/agencies to contact, and (6) ways to make the environment safe.

When reviewing the last step, the clinician asks about access to and availability of means, especially those that are part of a suicide plan. These include: firearms, pills or other ingestible poisons, sharp objects such as knives/scissors/razors, proximity to high places such as rooftops/bridges, and the opportunity for hanging or asphyxiation.

Step 5: Initiate coping strategies and supports

**Figure 2:**
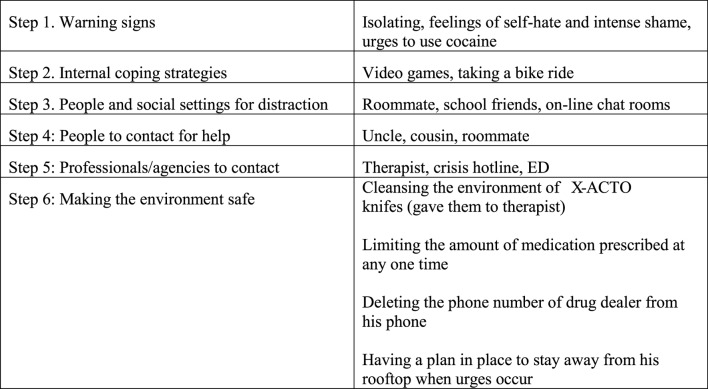
Paul’s Safety Plan.

The second step of the SPI is to generate a list of coping skills to use to manage suicidal urges independently. DBT distress tolerance skills for distraction and self-soothing (54) can be a helpful resource. See Figure 2 for Paul’s safety plan.

Step 6: Integrate suicide-specific treatment targets

Suicidal behavior is being increasingly understood as a symptom in its own right that needs to be specifically targeted in treatment (55). It is not enough to focus exclusively on non-suicidal treatment targets such as depressed mood or anxiety. Suicide–specific treatments (56) prioritize life threatening behaviors and offer a collaborative approach to engaging the patient in ongoing monitoring of suicidal thoughts, urges and behaviors. Paul’s treatment focuses explicitly on his suicidal ideation, urges and NSSI behaviors.

##### Monitor

Step 7: Increase flexibility and contact availability

The ZS model recommends provision of increased contact during periods of suicidal crises. This can take the form of increased number of appointments, and availability for between session check-ins by phone or e-mail. Following discharge from an inpatient or ED setting, follow-up phone calls or other forms of non in-person contact (e.g., letters; texts) can provide some sense of continuity of care. The AIM-SP model Structured Follow Up and Monitoring Intervention outlines the following process for making follow-up calls: 1. assessing mood and safety; 2. reviewing and revising the individual’s safety plan; 3. problem solving obstacles to follow-up care.

Step 8: Initiate increased monitoring during periods of highest risk

Knowing when to increase monitoring is key. Periods following a suicide attempt or suicide crisis, discharge from inpatient hospitalizations, an ED visit, transfer from higher to lower level of care, are well-known high-risk times. During care transitions, it is good practice to call other providers to give a “warm handoff”.

Step 9: Involve family and other social supports

With permission, a clinician can involve members of the individual’s support network to create a safety net. The clinician should obtain emergency contact information at initial contact, and encourage involving friends, family and other supports in treatment planning, means restriction and safety planning. Paul agreed to have his uncle and cousin involved in his care. They are a part of his safety plan and sometimes attend his therapy sessions. They help monitor Paul during high risk periods, check in with him regularly, and reach out to his clinician when necessary.

Step 10: Invoke clinician peer support and consultation

The clinician can also seek peer support for consultation and supervision regarding high risk patients. This includes maintaining contact and taking a team approach with other health providers involved with the patient, and reaching out when necessary to coordinate safety efforts.

## Summary

The ZS Initiative has been proposed by the United States National Alliance for Suicide Prevention and adopted by many health care systems in the US. We present the Assess, Intervene and Monitor for Suicide Prevention model (AIM-SP) to facilitate the implementation of the four clinical components, Identify, Engage, Treat, and Transition, of the ZS Model into an ongoing outpatient psychotherapy treatment. AIM-SP provides a framework for incorporating evidence-based and best suicide prevention approaches into clinical practice, and can inform training efforts to further disseminate evidence-based suicide prevention clinical practices. Future ZS efforts will include the application of this framework to other clinical and non-clinical medical settings, such as psychiatric inpatient services, psychiatric and medical EDs, primary care, and forensic settings.

## Author Contributions

BB: first author developed content, oversaw coauthor work, wrote majority of manuscript, and made final edits. AS-F: drafted and edited introduction and literature review, researched, and collated references. BS: developed and wrote AIM model and Safety Plan Intervention content, and made final edits.

## Conflict of Interest Statement

The authors declare that the research was conducted in the absence of any commercial or financial relationships that could be construed as a potential conflict of interest.
